# Pharmacological inhibition of demethylzeylasteral on JAK-STAT signaling ameliorates vitiligo

**DOI:** 10.1186/s12967-023-04293-2

**Published:** 2023-07-04

**Authors:** Yuqian Chang, Pan Kang, Tingting Cui, Weinan Guo, Weigang Zhang, Pengran Du, Xiuli Yi, Sen Guo, Tianwen Gao, Chunying Li, Shuli Li

**Affiliations:** Department of Dermatology, Fourth Military Medical University, Xijing Hospital, Xi’an, 710032 Shaanxi China

**Keywords:** Demethylzeylasteral, Vitiligo, CD8^+^ T cell, Keratinocytes, JAK, STAT

## Abstract

**Background:**

The activation of CD8^+^ T cells and their trafficking to the skin through JAK-STAT signaling play a central role in the development of vitiligo. Thus, targeting this key disease pathway with innovative drugs is an effective strategy for treating vitiligo. Natural products isolated from medicinal herbs are a useful source of novel therapeutics. Demethylzeylasteral (T-96), extracted from Tripterygium wilfordii Hook F, possesses immunosuppressive and anti-inflammatory properties.

**Methods:**

The efficacy of T-96 was tested in our mouse model of vitiligo, and the numbers of CD8^+^ T cells infiltration and melanocytes remaining in the epidermis were quantified using whole-mount tail staining. Immune regulation of T-96 in CD8^+^ T cells was evaluated using flow cytometry. Pull-down assay, mass spectrum analysis, molecular docking, knockdown and overexpression approaches were utilized to identify the target proteins of T-96 in CD8^+^ T cells and keratinocytes.

**Results:**

Here, we found that T-96 reduced CD8^+^ T cell infiltration in the epidermis using whole-mount tail staining and alleviated the extent of depigmentation to a comparable degree of tofacitinib (Tofa) in our vitiligo mouse model. In vitro, T-96 decreased the proliferation, CD69 membrane expression, and IFN-γ, granzyme B, (GzmB), and perforin (PRF) levels in CD8^+^ T cells isolated from patients with vitiligo. Pull-down assays combined with mass spectrum analysis and molecular docking showed that T-96 interacted with JAK3 in CD8^+^ T cell lysates. Furthermore, T-96 reduced JAK3 and STAT5 phosphorylation following IL-2 treatment. T-96 could not further reduce IFN-γ, GzmB and PRF expression following JAK3 knockdown or inhibit increased immune effectors expression upon JAK3 overexpression. Additionally, T-96 interacted with JAK2 in IFN-γ-stimulated keratinocytes, inhibiting the activation of JAK2, decreasing the total and phosphorylated protein levels of STAT1, and reducing the production and secretion of CXCL9 and CXCL10. T-96 did not significantly inhibit STAT1 and CXCL9/10 expression following JAK2 knockdown, nor did it suppress upregulated STAT1-CXCL9/10 signaling upon JAK2 overexpression. Finally, T-96 reduced the membrane expression of CXCR3, and the culture supernatants pretreated with T-96 under IFN-γ stressed keratinocytes markedly blocked the migration of CXCR3^+^CD8^+^ T cells, similarly to Tofa in vitro.

**Conclusion:**

Our findings demonstrated that T-96 might have positive therapeutic responses to vitiligo by pharmacologically inhibiting the effector functions and skin trafficking of CD8^+^ T cells through JAK-STAT signaling.

**Supplementary Information:**

The online version contains supplementary material available at 10.1186/s12967-023-04293-2.

## Background

Vitiligo is a common autoimmune skin disorder characterized by melanocyte destruction due to cytotoxic CD8^+^ T cells, resulting in hypo-pigmentary patches of skin all over the body or white hair [1]. Vitiligo affects approximately 0.5–2.0% of the population worldwide equally regardless of race, sex, or geography [2, 3]. Stacks of studies have shown that vitiligo is mostly a complex incorporation of abnormal immune responses, genetic predispositions, oxidative stress, metabolic alterations, and environmental factors [1, 2]. The treatment modalities for vitiligo have mostly been nontargeted and generalized like oral or topical immunosuppressants, phototherapy, and surgical methods with limited efficacy and potential side effects [4, 5]. There has been an emergence of innovative targeted therapies aimed at limiting disease progression and achieving repigmentation with a good safety profile based on a better understanding of the pathophysiology [6].

The exposure of autoantigens of melanocytes under various triggers initiates innate immune responses including antigen-presenting cells and NK cells, which subsequently activates the cytotoxic CD8^+^ T cell activation and leads to melanocyte destruction [7]. Additionally, the pro-inflammation factor interferon (IFN-γ) released by CD8^+^ T cells can induce the production of chemokines including C-X-C motif chemokine ligands 9 and 10 (CXCL9 and CXCL10) via JAK-STAT signaling in keratinocytes, fibroblasts or other skin cells [8, 9], which in turn can interact with their receptor C-X-C Motif Chemokine Receptor 3 (CXCR3) and promote CXCR3^+^CD8^+^ T cells recruiting to the melanocytes, exacerbating the progression of vitiligo [10, 11]. Accumulating studies have demonstrated that the inhibition of the effector functions and migration of CD8^+^ T cells is effective for vitiligo treatments [11–13].

Demethylzeylasteral (T-96), a bioactive triterpene product isolated from *Tripterygium wilfordii* Hook F (TwHF) in 1995 [14], possesses potent immunomodulatory and anti-inflammatory properties with less toxicity compared to other monomers of TwHF [15–17]. This has made it a promising small molecule candidate for future drug discovery. T-96 exhibits immunosuppressive effects in mouse splenocytes in vitro and in a rat kidney transplantation model in vivo [18, 19]. Furthermore, T-96 could reduce immunocompetent cells and inflammatory mediators in the rabbit model of atherosclerosis [19], inhibit inflammatory responses in mice with lupus nephritis [20–22], and suppress RNA-dependent RNA polymerase of hepatitis C virus [23]. Additionally, T-96 demonstrates anti-cancer abilities by inhibiting angiogenesis, cell proliferation, and cell apoptosis [15, 24–27]. Accumulating evidence suggests that T-96 may have the potential to be used in COVID-19 [28], arthritis [22], and other autoimmune disorders [22, 28] as a novel therapeutic agent. However, whether T-96 could make a difference in vitiligo treatment and the underlying mechanisms remain unreported.

Herein, the present study aimed to investigate the therapeutic efficacy of T-96 in the development of vitiligo using our mouse model and further explore the pharmacological inhibition of T-96 on the effector function and migration of CD8^+^ T cells from patients with vitiligo.

## Methods

### Patients and clinical samples

Fresh peripheral blood mononuclear cells (PBMCs) were collected from patients with diagnosed with vitiligo at active phase according to clinical manifestations and Wood’s lamp test in the Department of Dermatology of Xijing Hospital and healthy individuals at the Physical Examination Center of Xijing Hospital of the Fourth Military Medical University. 32 patients (3 for the determination of cell IC_50_ and cell viability of CD8^+^ T cells, 3 for target prediction, 6 for evaluating the proliferation and activation of CD8^+^ T cells, 8 for evaluating the effector function of CD8^+^ T cells, 6 for the migration of CD8^+^ T cells, 6 for the detection of chemokine receptors in CD8^+^ T cells) and sex- and age-matched 23 healthy volunteers (3 for the determination of cell IC_50_ and cell viability of CD8^+^ T cells, 6 for evaluating the proliferation and activation of CD8^+^ T cells, 6 for evaluating the effector function of CD8^+^ T cells, and 8 for the effects of T-96 upon JAK3 knockdown and overexpression) were recruited in this study. The research protocols for referring to human samples were performed according to the principles of the Declaration of Helsinki. The study was approved by the Ethics Committee of the Fourth Military Medical University, and written informed consent was obtained from all participants.

### Induction of vitiligo mice and treatment

All animal studies were reviewed and approved by the Ethics Committee of Animal Care of the Fourth Military Medical University (FMMU). All animal experiments were conducted according to the guidelines for animals. Specific pathogen-free (SPF) female C57BL/6 mice (8–10 weeks old) were obtained from the Laboratory Animal Center of FMMU. All mice were housed in micro isolator cages in an SPF setting at 24 ± 2°C and exposed to a 12 h light/12 h dark cycle, with standard feed and water provided ad libitum at the Experimental Animal Center of FMMU.

C57Bl/6 mice were intradermally injected with 2 × 10^5^ B16F10 cells (B16 melanoma cell line of C57BL/6 mouse origin) in 0.1 ml PBS per mouse (cell viability was more than 96% characterized by harvesting after limited passage in vitro and off-white to the white color of cell mass after centrifugation) into the left-back on day 0 and then treated with anti-mouse CD4 antibody (clone, GK1.5, Biolgend) intraperitoneal (i.p.) injection on day 4 and day 10 at 10 mg/kg (only mice with a tumor larger than 1 mm in diameter on day 4). CD4^+^ T cell preferential depletion from mice with advanced melanoma can activate CD8^+^ T cells against B16 melanoma cells resulting in the destruction of the melanocytes in hair bulbs due to cross-antigenicity between both cell types [29]. On day 12, primary tumors were surgically excised from the skin and the incision was closed (performed on mice with no subcutaneous infiltration). Mice were monitored every other day to ensure no tumor metastases and recurrent primary tumors, and 85% of mice showed vitiligo clinical signs after 40–50 days. The extent of depigmentation was objectively quantified with the percentage of the anatomic site (tail) using Image J software (images converted to 8-bit black on pigmented areas and white on depigmented areas).

Treatments were administrated on day 28 (CD8^+^ T cells initial infiltration usually began at day 24 to day 28) based on the continuous observation of CD8^+^ T cell presence in the epidermis using whole-mount tail epidermis staining at different periods. To identify the optimal treatment dose of T-96, we used two doses 2.5 mg/kg and 5.0 mg/kg referencing the treatment in a mouse model of breast cancer [25] and the melanoma mouse model [30]. The vitiligo mice were randomly divided into five groups (T-96 at 2.5 mg/kg and 5 mg/kg, Tofa at 5 mg/kg, T-96 combined with Tofa at 5 mg/kg, and vehicle) before treatment. T-96 and Tofa were first dissolved in a small volume of Dimethyl sulfoxide (DMSO, Thermo Fisher Scientific) and later diluted with sterile PBS and then stored according to the manufacturers’ suggestions. T-96 and Tofa treatment were performed by intraperitoneal injection once every other day for five weeks.

### Epidermal Whole-mount staining and imaging

The tails were depilated with Nair™, and tail skin was stripped from the tailbone and flattened in a 12-well plate (trimmed into 1 × 1 cm pieces) for 5 min. Then, the plate was incubated with 1 mL 20 mM EDTA (pH 8.0) solution at 37 °C. After 2 h, carefully obtained the epidermis from the dermis with a fine-tipped tweezer in the anterior–posterior direction under the stereomicroscope (also removed the hair follicles and sebaceous gland), flattened and fixed the epidermis in 4% paraformaldehyde for 10 min and further immersed with 0.3% H_2_O_2_/methanol for 20 min at − 20 °C whereafter washed with PBS for 3 times (15 min per time at room temperature). Blocked in a solution of 2% donkey serum, 1% BSA and 0.3% TritonX-100 for 1 h with shaking, then incubated with primary antibodies (anti-CD8a, and anti-MelanA) (Additional file 2: Table S1) in blocking buffer overnight at 4 °C on a shaker. Secondary antibodies (Goat-anti-Rat FITC and Goat-anti-mouse Cy3) (Additional file 2: Table S1) with DAPI (0.2ug/ml) in blocking solution were added for 8 h at 4 °C on a shaker. Finally, the samples were mounted onto slides using glycerin and DAPI and secured with cover glass. We removed hair follicles and sebaceous glands due to strong non-specific green fluorescence staining with interfering CD8^+^ T cells for the following experiments. Whole-mount tail epidermis images were acquired in a sequential manner using the sequential scan mode using FV-1000/ES confocal microscope (Olympus, Tokyo, Japan), setting Z-stack to ensure that the z-position covers the entire thickness of samples. Images were exported using ZEN 2012 (Carl Zeiss Microscopy GmbH), optimized globally for color balance, brightness, and contrast using Photoshop CC 2018 (Microsoft). Measurements of the numbers of CD8^+^ T cells and melanocytes were performed using ImageJ software.

### ***CD8***^+^***T cells isolation and proliferation assay***

PBMCs were isolated using Ficoll-Hypaque density gradient centrifugation (Dakewei, Shenzhen, China). CD8^+^ T cells were purified from PBMCs using the CD8^+^ T Cell Isolation Kit (Miltenyi, USA) according to the manufacturer’s instructions, and the purity of CD8^+^ T cells was more than 95% monitored by flow cytometry using anti-CD3 and anti-CD8 (Additional file 1: Fig. S1A). CD8^+^ T cells were cultured in complete RMPI medium that contained RPMI 1640 (GlutaMAX™, Gibco, USA) supplemented with 10% fetal bovine serum (FBS, Gibco, USA), penicillin (100 U/ml), streptomycin (0.1 mg/ml) (all from Invitrogen, USA). For proliferation assay, CD8^+^ T cells were stained using the carboxyfluorescein succinimidyl ester (CFSE, Thermo Fisher Scientific, USA). Briefly, purified CD8^+^ T cells were washed with PBS and incubated with CFSE dye at a final concentration of 5 μM for 15 min at 37 °C protected from light. Next, the complete RMPI medium was added to the cell suspension and incubated for 5 min before being washed and then resuspended in complete RPMI medium. CFSE-labeled CD8^+^ T cells were cultured in 96 well plates and stimulated with dynabeads human T-activator CD3/CD28 at 1:200 (Thermo Fisher Scientific, USA) in the presence of hIL-2 (R&D, USA) at 20 ng/ml. Demethylzeylasteral (T-96) (Solarbio Science, CAS No. 107316-88-1, China) or Tofacitinib (Tofa) (MedChem Express, CAS No. 477600-75-2, USA) were stimulated both at 1 µM. After 5 days, the proliferation levels were analyzed using flow cytometry.

### ***CD8***^+^***T cells stimulation and flow cytometry***

For the evaluation of activation and effect function, CD8^+^ T cells were stimulated with plate-bound anti-CD3 (2 μg/ml, BioLegend, USA), plate-bound anti-CD28 (1 μg/ml, BioLegend, USA), and hIL-2 (20 ng/ml) in complete RPMI medium. T-96 and Tofa were treated according to the experimental design. After indicated times, cells were harvested for analysis, and a cocktail of Brefeldin A and Monensin (eBioscience, USA) was added to suppress cytokine release at 6 h before intracellular cytokine staining. To investigate the JAK-STAT signaling pathway, CD8^+^ T cells were pretreated with T-96 and Tofa for 1 h followed by IL-2 stimulation. For surface marker staining, cells were stained with antibodies (anti-CD69 and anti-CXCR3) (Additional file 2: Table S1) diluted at a final concentration 1 μg/mL and human Fc Receptor Blocker (BD, USA) to inhibit nonspecific antibody binding at 1:50 ratio in FACS buffer (PBS, 1%BSA, 0.1% NaN3 sodium azide), then incubated at 4 °C in the dark for 30 min. Isotype control antibodies (Additional file 2: Table S1) were used to determine the background caused by nonspecific antibody binding. Next, samples were washed twice using FACS buffer, suspended with FACS buffer with propidium iodide (1:100), and analyzed with the CytomicsTM FC500 flow cytometer (Beckman Coulter) in 30 min.

For intracellular staining, single-cell suspensions were stained with Fixable Viability Stain 620 (BD, USA) for 15 min at room temperature. The cells were then fixed and permeabilized with Transcription Factor Staining Buffer Set (Invitrogen) according to the manufacturer’s instructions and stained with antibodies (anti-IFN-γ, anti-GzmB, anti-PRF, anti-p-STAT5 (pY694) and anti-p-JAK3 (Tyr980/981)) (Additional file 2: Table S1) in prepared FACS buffer (supplemented with human Fc Receptor Blocker) for 1 h at room temperature. Samples were then analyzed in 48 h using CytomicsTM FC500 flow cytometer (Beckman Coulter, USA) and data were analyzed using FlowJo software.

### Cell culture, stimulation, and plasmid transfection

Primary normal human epidermal keratinocytes (NHEKs) were isolated from the epidermis of plastic surgery skin (healthy subjects, 8 to12 years), then cultured in EpiLife™ medium (Gibco, USA) plus with keratinocyte growth supplement (Gibco, USA). Second to fifth passage NHEKs were used, and each experiment was repeatedly performed with at least three different donors. HaCaT cells (the human keratinocyte cell line) were cultured in RPMI 1640 supplemented with 10% FBS. Mouse melanoma cell line B16F10 was cultured in a complete Dulbecco’s Modified Eagle Medium (DMEM, high glucose, Thermo Fisher Scientific, USA) with 10% FBS. The two cell lines were authenticated by short tandem repeat (STR) fingerprinting by Fourth Military Medical University and showed no mycoplasma contamination. All cell mediums contained penicillin (1000 U/ml), streptomycin (1 mg/ml) (all from Invitrogen, USA). All cells were cultured at 37 °C in a humidified incubator with 5% CO_2_.

Recombinant human IFN-γ at 20 ng/ml (Peprotech, USA), T-96 at indicated concentration, and Tofa at 1 µM was performed in NHEKs and HaCaT cells. Plasmids for knockdown targeting JAK2 (sc-39099) and JAK3 (sc-29379) were purchased from Santa Cruz. Plasmids for overexpression targeting JAK2 and JAK3 were synthesized by Tsingke (Tsingke, China). Empty plasmids were used as negative controls. Cells were transfected using the Lipofectamine 3000 transaction Reagent kit (Invitrogen, USA) according to the manufacturer’s instructions.

### Cytotoxicity assessment using CCK-8 assay

The cell viability index was measured using the cell counting kit-8 (CCK-8) (Beyotime Biotechnology, China). Briefly, cells were seeded in 96-well plates and stimulated with T-96 at a series of expected concentrations for 5 days for the IC_50_ detection and 48 h for cell viability. Then CCK-8 reagent (diluted 1:10 in 100 μL fresh medium) was added to each well, and the plates were incubated for another 90 min. The plate was then read with a Model 680 Microplate Reader (Bio-Rad, Hercules, CA, USA) at an absorbance reading of 450 nm. A logistic regression model for the calculation of the inhibitory concentration 50% (IC_50_) to determine the cytotoxic activity of T-96.

### Pull-down assay

T-96^Bio^ (biotinylated T-96) was synthesized, analyzed by mass spectrometry and HPLC, and stocked at −20 °C after a freeze-drying process. CD8^+^ T cells or NHEKs (stimulated with IFN-γ) were lysed with a complete lysis buffer that contained NP-40 (Beyotime, China) with protease and phosphatase inhibitor cocktail (Sigma-Aldrich). To obtain the target protein of T-96, we first incubated T-96^Bio^ and the prewashed (with complete lysis buffer) M-280 Streptavidin Dynabeads (Invitrogen, Cat.11205D, USA) at room temperature for 30 min in a shaker and placed the tube into a magnetic stand to collect the T-96^Bio^-beads mix, then T-96^Bio^-beads were incubated with the cell protein lysates for each sample overnight at 4 °C in a shaker to obtain the T-96^Bio^-beads-protein compound (washed twice with complete lysis buffer to avoid the non-specific binding protein). All collected T-96^Bio^-beads-protein complexes were eluted with protein loading buffer at 100 °C for 5 min and stored at -80 °C for Mass Spectrum analysis.

### Mass spectrum and gene ontology analysis

T-96^Bio^-beads-protein samples were subjected to SDS-PAGE (10% gels), and then gels were stained with Coomassie blue G250 (BIO-RAD, Cat.1610406, USA). The protein bands of interest were excised from Coomassie blue-stained gel, diced into small pieces, and destained by washing twice with 25 mM NH_4_HCO_3_/30% acetonitrile. All Samples were prepared according to the standard protocol as described previously [31], and finally dissolved with 2% acetonitrile/98% H2O/0.1% TFA for mass spectrometry analysis using the QExactive system (Thermo Fisher Scientific, USA). The protein identification and quantitation analysis were performed using Protein Pilot software (AB Sciex, USA). Gene Ontology (GO) biological process analysis was performed using Metascape. Dot plots were plotted using http://www.bioinformatics.com.cn, a free online platform for data analysis and visualization.

### Molecular docking

A study of in silico docking of T-96 with JAKs was conducted as described previously [32]. In brief, the binding regions and the key amino acids of JAK3, JAK2, and JAK1 were defined according to the published literature and the PDB database. Molecular structures were energy optimized for molecular docking using the Prepare Ligands module present in the Discovery Studio 3.5 (Accelrys Inc.) and converted to the SD file format. The parameters for docking were determined by analysis of poses obtained by docking JAK1(PDB code: 4EHZ), JAK2 (PDB code: 5UT0), and JAK3 (PDB code: 5TOZ), which were downloaded from Protein Data Bank in PDB format. The number of generated poses was set to 100 for each protein, and default settings were selected for other parameters. Before docking, the original crystal and water molecules were removed from the T-96-protein complexes. Hydrogen atoms were added via the application of CHARMM force field and the Momany-Rone partial charge default settings in Discovery Studio 3.5. Docking analyses of T-96 with JAKs were performed by means of the CDOCKER module, which is accurate when active sites are known. This method meets the requirements of experimental verification.

### Quantitative polymerase chain reaction assay

Total RNA was isolated using Trizol reagent (Invitrogen, USA) based on the manufacturer's instruction, then quantified with the Multiskan spectrophotometer (Thermo Scientific, USA). The cDNA synthesis was performed using the PrimeScript RT reagent Kit (Takara, Japan). Real-Time Quantitative Reverse Transcription PCR (RT-qPCR) assays were performed in iQ5 Real-Time PCR Detection System (Bio-Rad) using SYBR Premix Ex Taq II (TaKaRa) to determine the expression of mRNA. The primers used are listed in Additional file 2: Table S2. Samples were analyzed in triplicate and normalized to ACTIN.

### Western blot

After indicated treatment, total protein was obtained from cells with cell lysis buffer RIPA (Beyotime, China) supplemented with protease and Phosphatase Inhibitor (Sigma-Aldrich, USA), and then quantified using BCA protein assay kit (Beyotime, China). Equal amounts of protein were run on 10% SDS-PAGE (Bio-Rad) and blotted into polyvinylidene difluoride membranes (Millipore, Billerica, USA). After blocking in a solution of 5% fat-free dried milk diluted in Tris-buffered saline, the membranes were incubated with primary antibodies (anti-JAK2, anti-p-JAK2 (Tyr1007/1008), anti-STAT1, anti-p-STAT1 (Tyr701), anti-β-actin) (Additional file 2: Table S1) overnight at 4 °C, and then with horseradish peroxidase-conjugated anti-rabbit or anti-mouse secondary antibodies (Additional file 2: Table S1) at 1:5000 for 1 h at room temperature. Signals were visualized with the ECL western blotting detection system (Millipore, USA) and Image J software (Bio-Rad).

### Immunofluorescence staining

NHEKs were grown and treated in single-layer glass slides as previously reported [33]. After washing and fixing, cells were incubated with primary antibodies from Cell Signaling (anti-p-STAT1 and anti-STAT1) (Additional file 2: Table S1) overnight. Then cells were subsequently incubated with the secondary antibodies Goat-anti-Rabbit IgG cy3 and goat anti-Mouse IgG H&L FITC (Additional file 2: Table S1) for 1 h and further the nuclear dye (DAPI) for 15 min at room temperature. Fluorescent images were obtained using an FV-1000/ES confocal microscope (Olympus, Tokyo, Japan).

### ELISA assay

Cell-free supernatants from stimulated NHEKs (10^6^ cells per mL) were tested. The production of chemokines was detected using ELISA kits CXCL9 (Elabscience, Wuhan, China), and CXCL10 (R&D, USA) according to the corresponding manufacturer’s instructions.

### The transwell migration assay

NHEKs were pretreated with T-96, Tofa, T-96 combined with Tofa, and T-96 combined with JAK2 knockdown or overexpression following the IFN-γ stimulation. After 48 h, culture supernatants were collected for the following migration assay. CXCR3^+^CD8^+^ T cells (Additional file 1: Fig. S1B) sorted from PBMC of patients with vitiligo were seeded in the upper chamber (1 × 10^5^ cells cultured in 100 μl complete RPMI 1640 medium) and separated from the cell supernatants in the lower chamber by using a 5.0 μm polycarbonate membrane (Corning, USA). Additionally, neutralization antibodies of CXCL9 (MAB392; R&D, USA) and CXCL10 (MAB266; R&D, USA) were added at 10 ng/mL to block the chemokines CXCL9 and CXCL10 in the culture supernatants. Plates were maintained at 37 °C for 3 h, and then migrated cells in the lower chamber were counted by means of flow cytometric analysis acquisition thereafter. Migration of CXCR3^+^CD8^+^ T cells was calculated as the percentage of cells migrating from the upper to the lower chamber.

## Statistical analyses

Data analysis was performed using GraphPad Prism version 9.0 software (GraphPad Software, San Diego, CA, USA). All experiments were repeated at least three times unless. Error bars presented as mean ± SD. Dual comparisons were made with two-tailed Student *t* test. Groups of three or more were analyzed by one-way analysis of variance (ANOVA) with Dunnett’s test. *P* values less than 0.05 were considered statistically significant.

## Results

### ***T-96 ameliorated ongoing depigmentation and inhibited the CD8***^+^***T cell skin infiltration in our mouse model of vitiligo***

**Fig. 1:**
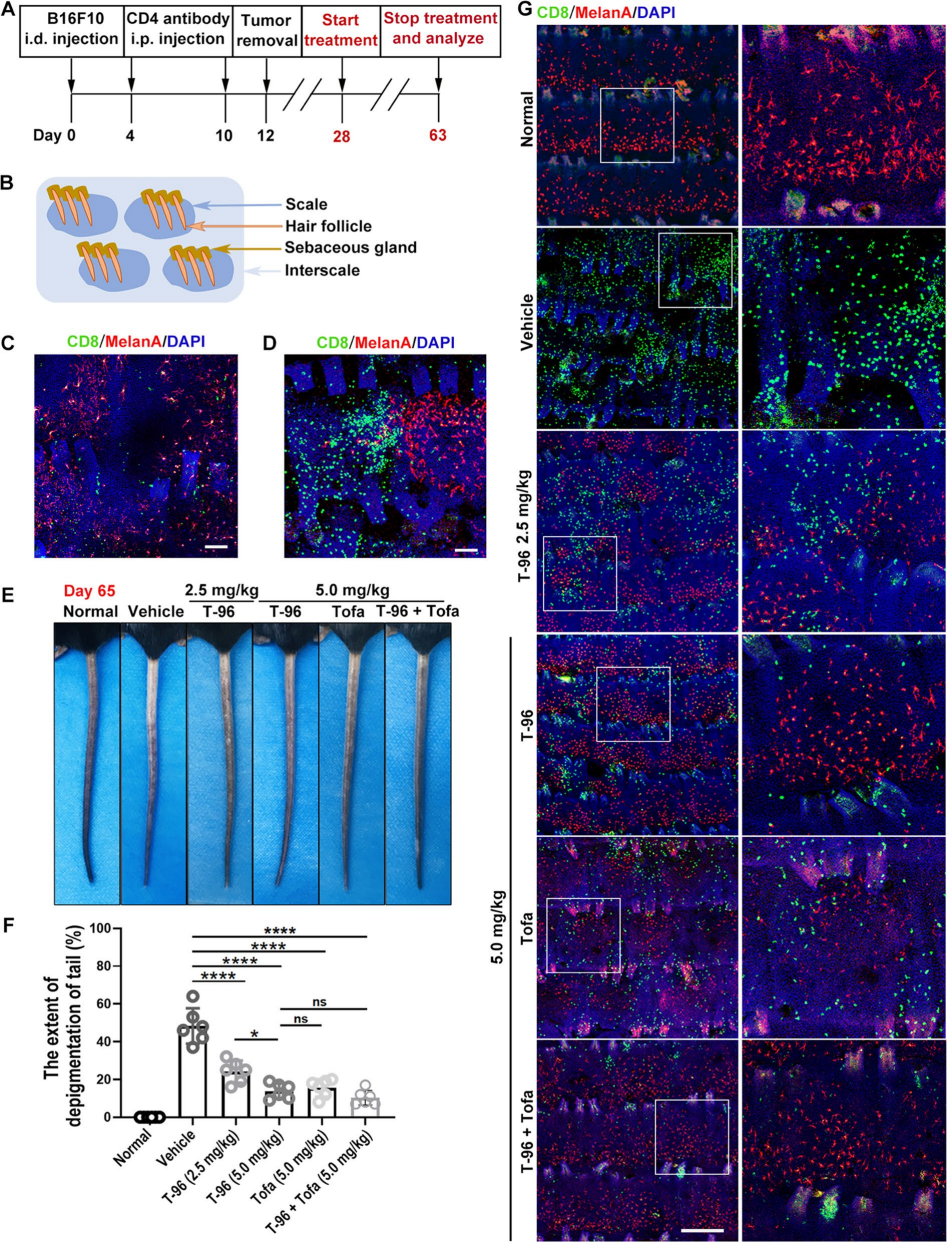
T-96 ameliorated ongoing depigmentation by inhibiting the CD8^+^ T cells skin infiltration in a mouse model of vitiligo. **A** Schematic diagram of vitiligo induction and treatment. **B** Schematics of the whole-mount view of tail skin. **C** Initial accumulation of CD8^+^ T cells around follicles. Scale bar = 100 μm. **D** Strong accumulation of CD8^+^ T cells in close vicinity to melanocytes. Scale bar = 100 μm. **E** The representative mouse tail images. **F** Statistical analysis of the extent of pigment loss. **G** Representative whole-mount tail epidermis immunofluorescence staining at indicated treatments for five weeks. Scale bar = 200 μm. Three replicate experiments were performed for a total of 6 mice per group. *P < .05, ****P < .0001. *ns* not significant

To address the treatment effect of T-96 in vitiligo we constructed our mouse model as described in Methods and summarized in Fig. 1A, which relied on endogenous auto-reactive CD8^+^ cell targeting melanocytes achieved through transient inoculation of B16F10 melanoma cells and depletion of CD4^+^ regulatory T cells. The whole-mount staining of the normal tail epidermis of C57BL/6 mice is shown in Additional file 1: Fig. S2A and a schematic of the whole-mount view is shown in Fig. 1B. By using whole-mount tail epidermis staining, we observed the epidermis infiltration of CD8^+^ T cells on day 28 (Fig. 1C), and then CD8^+^ T cells migrated to the entire scale accompanied with melanocyte-specific elimination (Fig. 1D), indicating high concordance with the pathologies of human vitiligo and the feasibilities of translation studies and drug discovery efforts.

To clarify the immunosuppressive action of T-96 in vitiligo, tofacitinib (Tofa) was used as a positive control, in that Tofa could induce successful repigmentation in vitiligo patients [34] and suppress CD8^+^ T cells function in our previous study [35]. We began treatment on day 28 with T-96 at two doses (2.5 mg/kg and 5.0 mg/kg) intraperitoneal injection once every other day for five weeks. The results showed that T-96 reduced depigmentation compared to vehicle-treated controls, and the mice treated with 5.0 mg/kg had a better response than the mice with 2.5 mg/kg (Fig. 1E, F). Also, we found that either T-96 or Tofa treated mice at 5 mg/kg achieved competent improvement and combined treatment of both had minor cooperativity but no statistical difference (Fig. 1E, F). Next, we quantified the number of CD8^+^ T cells and melanocytes using whole-mount staining and observed that the mice treated with T-96 or Tofa exhibited less CD8^+^ T cell invasion and more melanocytes remaining compared with that of the vehicle group (Fig. 1G and Additional file 1: Fig. S2B, C). These results suggested that T-96 could improve depigmentation by diminishing CD8^+^ T cell skin infiltration in the mouse model of vitiligo.

### ***T-96 suppressed the proliferation, activation, and function of CD8***^+^***T cells***

**Fig. 2:**
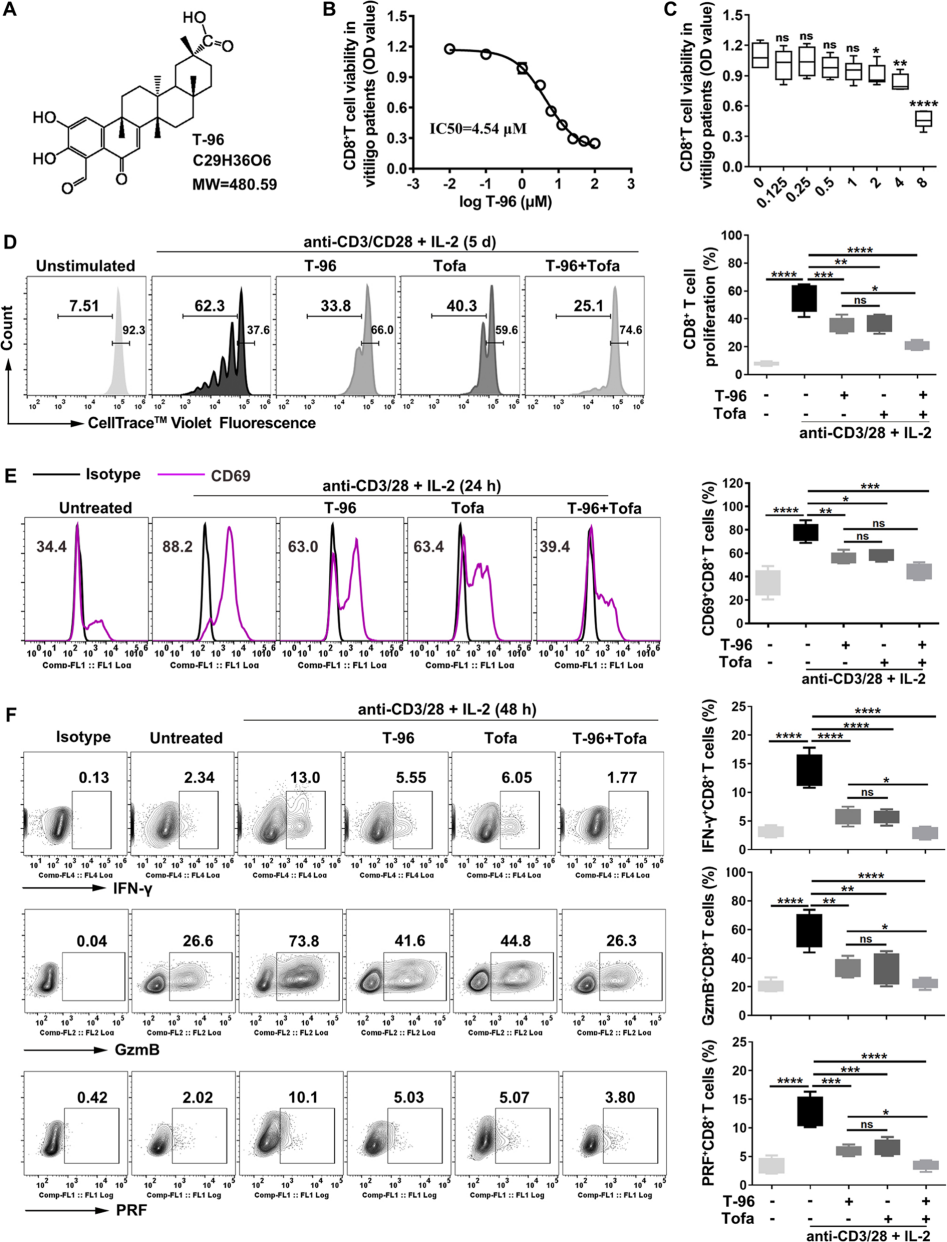
T-96 suppressed the proliferation, activation, and function of CD8^+^ T cells. **A** Structure of T-96. **B**, **C** CD8^+^ T cells were cultured under anti-CD3/CD28 Abs, IL-2 and T-96 for indicated dose for IC_50_ value and cell viability. Statistical analysis was performed relative to the untreated group. **D** CFSE-labeled CD8^+^ T cells were treated with T-96, Tofa, or both in the presence of IL-2 and anti-CD3/CD28 beads. Representative histograms of proliferation (left) and statistical graph (right). **E**, **F** Flow cytometry for the expression of CD69, IFN-γ, GzmB and PRF, representative examples (left), and frequency analysis (right). *P < 0.05, **P < 0.01, ***P < 0.001, ****P < 0.0001, *ns* not significant

The chemical structure of T-96 was depicted in Fig. 2A. The 50% inhibitory concentration (IC50) value of T-96 was 4.54 μM and 6.32 μM in CD8^+^ T cells isolated from the peripheral blood of vitiligo patients and healthy controls respectively (Fig. 2B and Additional file 1: Fig. S3A). 1.0 μM was applied for the non-toxic and effective dosage for subsequent experiments based on cytotoxicity assessment (Fig. 2C and Additional file 1: Fig. S3B). To investigate the immunosuppressive action of T-96, we pretreated CD8^+^ T cells with interleukin-2 (IL-2), which is an important γ chain cytokine regulating the proliferation and function of CD8^+^ T cell [36]. The CFSE flow cytometry results showed that both T-96 and Tofa suppressed CD8^+^ T cell proliferation in patients with vitiligo (Fig. 2D) as well as in healthy individuals (Additional file 1: Fig. S3C), and the combination of T-96 and Tofa showed stronger suppressive proliferation action in vitiligo patients (Fig. 2D). Next, we found that the frequency of early activation marker CD69 on CD8^+^ T cells was decreased when treated with T-96 or Tofa in the presence of IL-2 for 24 h, and there was a dim collective effect but statistically insignificant when two molecules co-pretreated from both patients with vitiligo (Fig. 2E) and healthy individuals (Additional file 1: Fig. S3D). Subsequently, we evaluated the effector function of T-96 in CD8^+^ T cells and revealed that T-96 decreased the high frequencies of pro-inflammatory cytokine IFN-γ, as well as cytotoxic-associated GzmB and PRF like the effect of Tofa in both the patients and healthy individuals, and joint administration of both demonstrated mild synergistic action in vitiligo patients (Fig. 2F), but statistically insignificant in healthy controls (Additional file 1: Fig. S3E). Together, comparable with Tofa, T-96 could suppress the proliferation, activation, and cytotoxic function of activated CD8^+^ T cells, indicating the potential inhibitory effect of autoreactive CD8^+^ T cells in the pathogenesis of vitiligo.

### ***T-96 inhibited JAK3-STAT5 signaling in CD8***^+^***T cells***

**Fig. 3:**
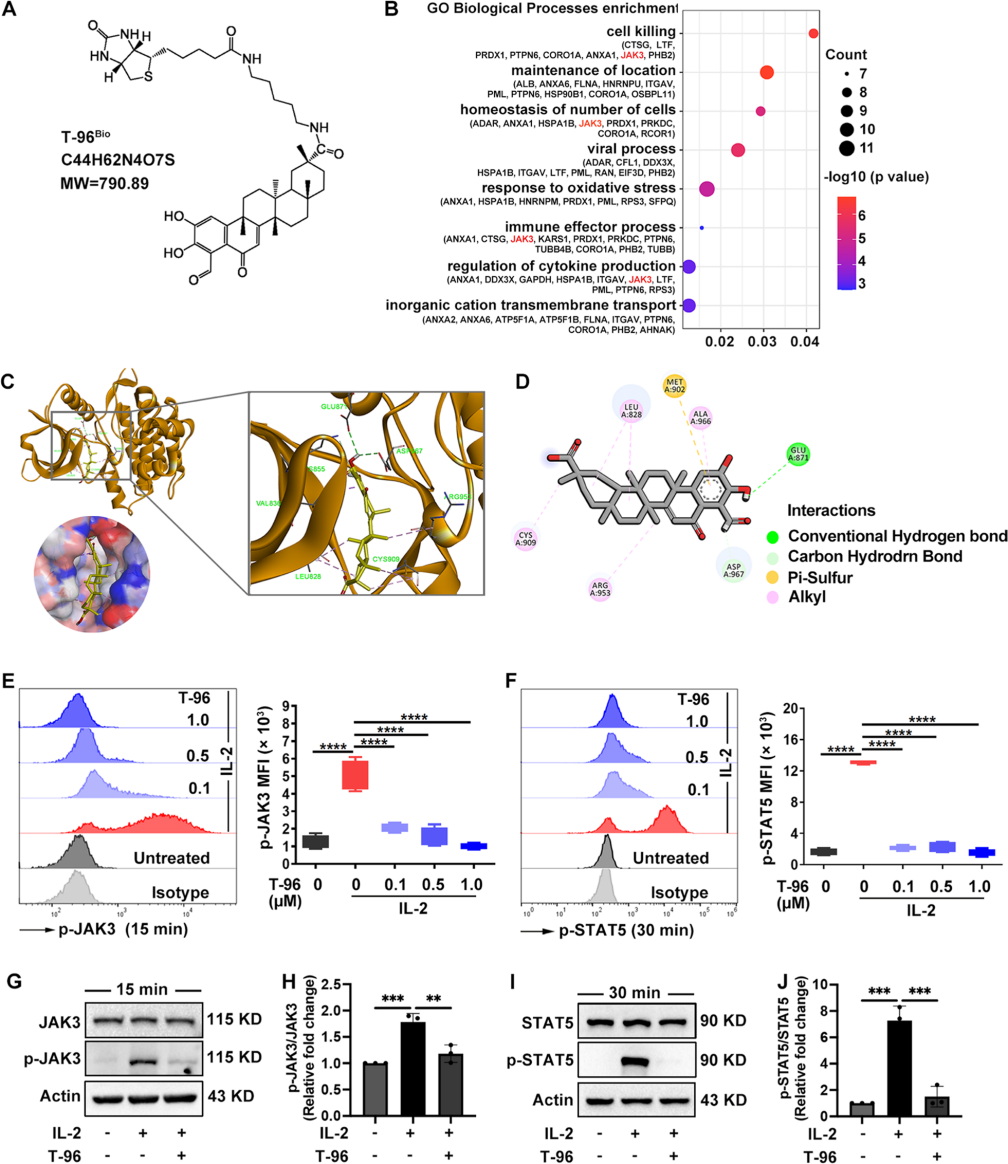
T-96 inhibited JAK3-STAT5 pathway in CD8^+^ T cells. **A** The structure of T-96^Bio^. **B** The enriched GO biological process analysis of binding proteins with T-96 ^Bio^ in CD8^+^ T cells. **C** Molecular docking analysis of T-96 (chartreuse stick) interacting JAK3 kinase domain (brown, PDB: 5TOZ) at the top and the solvent accessible surface area at bottom. **D** Interactions were colored by type. **E**, **F** The expression of phosphorylated JAK3 (p-JAK3) at 15 min and phosphorylated STAT5 (p-STAT5) at 30 min were presented as representative plots (left) and the mean fluorescence intensity (MFI) statical analysis (right). **G**, **H** The protein expression of JAK3 and p-JAK3 at 15 min and the ratios of p-JAK3/JAK3. **I**, **J** The protein expression of STAT5 and p-STAT5 at 30 min and the ratios of p-STAT5/ STAT5. **P < 0.01, ***P < 0.001, ****P < 0.0001

We next investigated the molecular mechanism underlying the impairment function in CD8^+^ T cells of T-96. Firstly, to identify the potential binding proteins of T-96 in CD8^+^ T cells, we synthesized biotinylated T-96 (T-96^Bio^, Fig. 3A) and obtained the streptavidin bead-T-96^Bio^-protein complexes from CD8^+^ T cells total lysates by pull-down assay. Isolated T-96 binding proteins were further identified by mass spectrometry analysis and suggested T-96 participated in immune effector processes and the regulation of cytokine production (Fig. 3B). Notably, JAK3 was one of the binding proteins in CD8^+^ T cells (Fig. 3B). Thus, we performed computational structure prediction for docking simulation and found T-96 docked into the JAK3 kinase domain (Fig. 3C), forming a conventional hydrogen bond with Glu871, carbon-hydrogen bond with Asp967, Pi-sulfur interaction with Met902, and alkyl interactions with Leu828, Cys909, Ala966, and Arg953 (Fig. 3D). We subsequently assessed the effects of T-96 on JAK3 and STAT5 phosphorylation of in CD8^+^ T cells using flow cytometry. We found that pretreatment with T-96 for 1 h reduced p-JAK3 (Fig. 3E) and p-STAT5 (Fig. 3F) expression in a dose-dependent manner. Moreover, western blot analysis revealed that compared with the IL-2 treated group, treatment with T-96 also reduced p-JAK3 (Fig. 3G) and p-STAT5 (Fig. 3I) expression and decreased the ratios of p-JAK3/JAK3 (Fig. 3H) and p-STAT5/STAT5 (Fig. 3J). These data demonstrated that T-96 suppressed the JAK3-STAT5 signaling in IL-2-activated CD8^+^ T cells from patients with vitiligo.

**Fig. 4:**
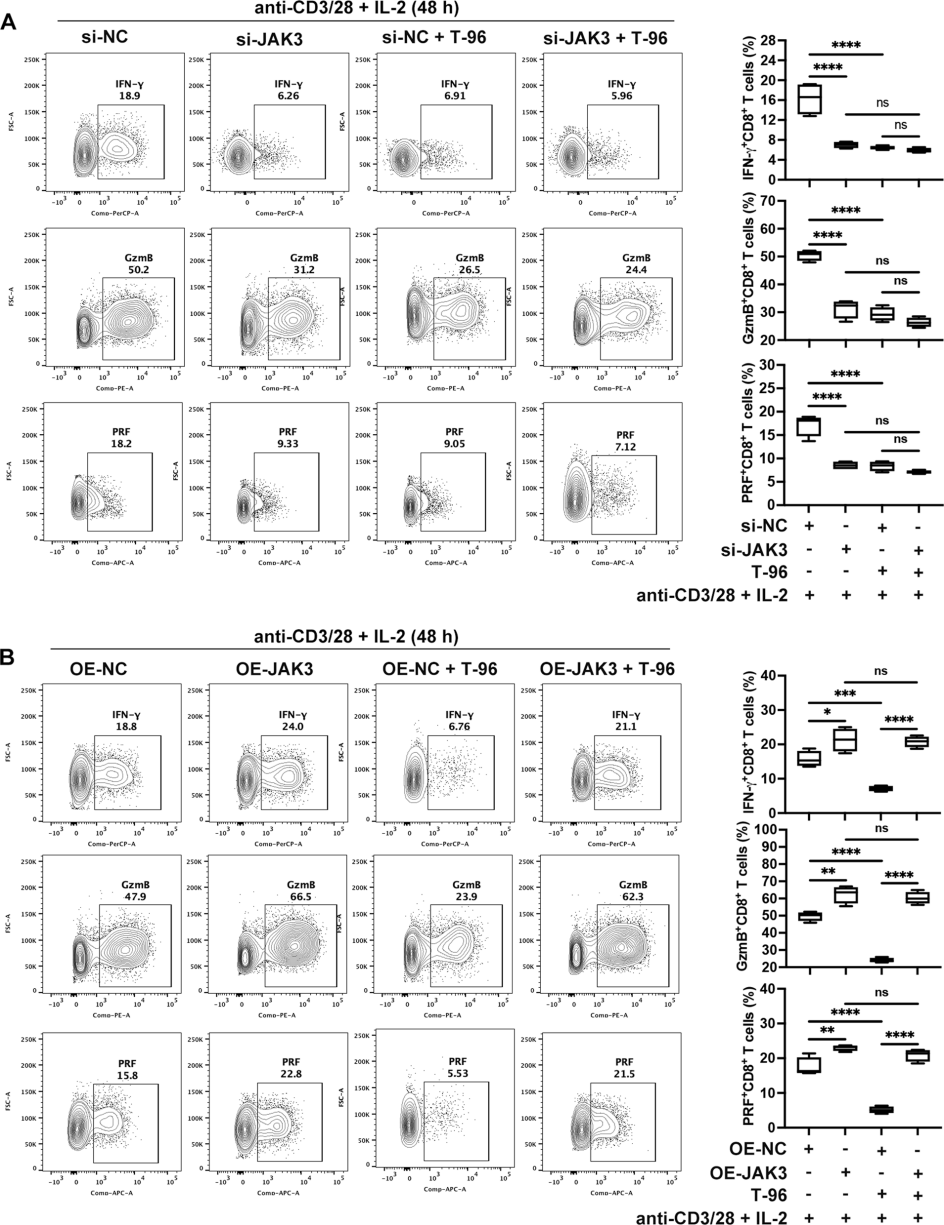
T-96 exerted immunosuppressive effects primarily by targeting JAK3. **A** Flow cytometry analysis of IFN-γ, GzmB and PRF expression upon JAK3 knockdown, representative examples (left), and frequency analysis (right). **B** Flow cytometry for the expression of IFN-γ, GzmB and PRF upon JAK3 overexpression, representative examples (left), and frequency analysis (right). *P < 0.05, **P < 0.01, ***P < 0.001, ****P < 0.0001, *ns* not significant

Next, we assessed the effect of T-96 on CD8^+^ T cells with JAK3 knockdown or overexpression. The efficiency of JAK3 knockdown and overexpression was shown in Additional file 1: Fig. S4A, B. The results showed that knocking down JAK3 significantly reduced the expression of the IFN-γ, GzmB and PRF, and pretreatment with T-96 did not further diminish these immune effectors in JAK3 knockdown group compared to the blank control plasmid group (Fig. 4A). In addition, JAK3 overexpression upregulated the expression of IFN-γ, GzmB and PRF to some extent in the presence of IL-2. Notably, pretreatment with T-96 was unable to inhibit the increased expression of these immune effectors in the JAK3 overexpression compared to the blank control plasmid group (Fig. 4B). Collectively, these results suggested that T-96 exerted immunosuppressive effects primarily by targeting JAK3.

### *T-96 decreased the expression of CXCL9/10 *via* JAK2-STAT1 pathway in IFN-γ stressed keratinocytes*

**Fig. 5:**
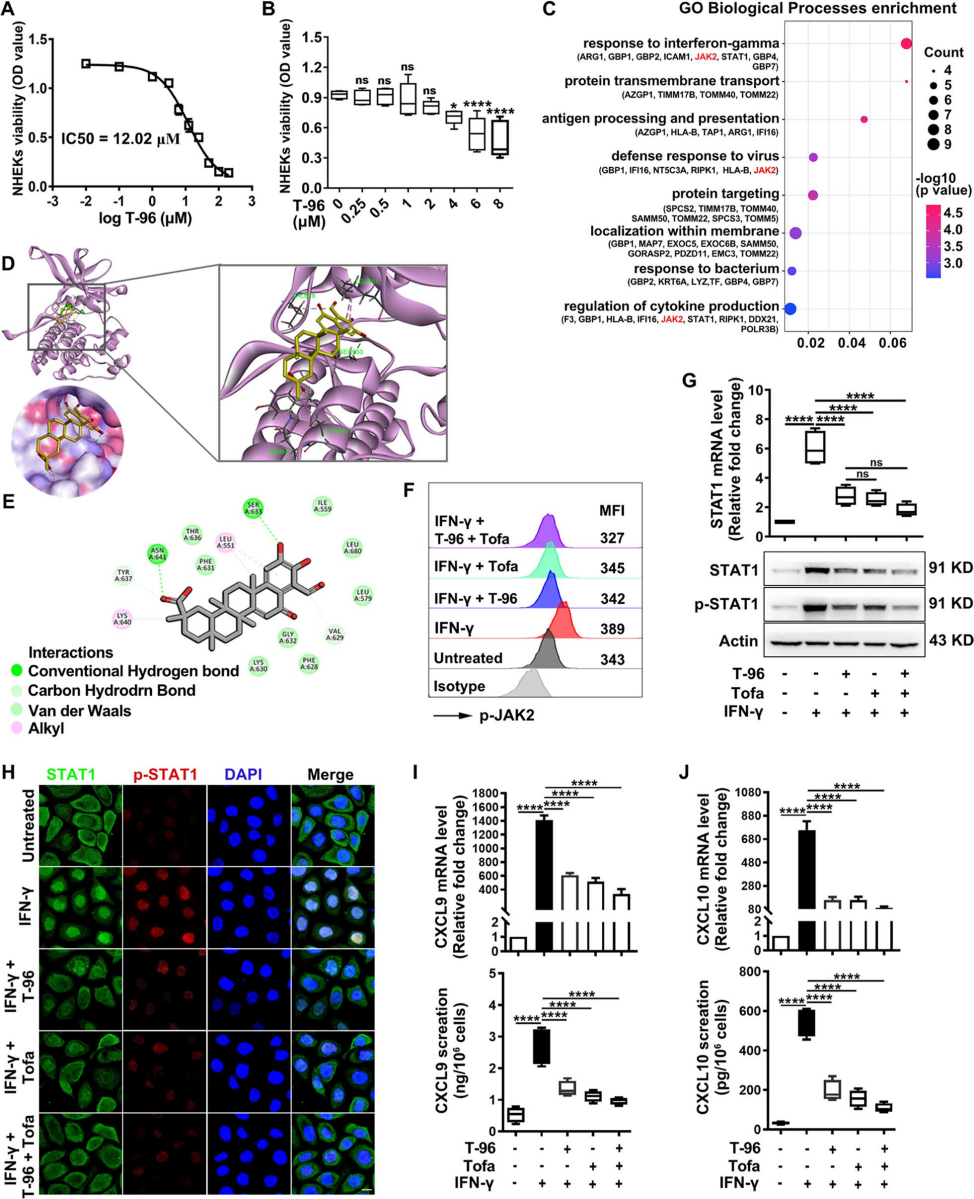
T-96 decreased the expression of CXCL9/10 via JAK2-STAT1 pathway in IFN-γ treated keratinocytes. **A**, **B** IC_50_ value and cell viability of T-96 in NHEKs. Statistical analysis was performed relative to the untreated group. **C** The GO analysis of the binding proteins of T-96 in IFN-γ-stimulated NHEKs. **D** Docking analysis of T-96 (chartreuse stick) with JAK2 kinase domain (purple, PDB: 5UT0) (top) and the solvent accessible surface area (bottom). **E** Interactions were colored by type. **F** Representative histogram of p-JAK2. **G** The mRNA and protein expression of STAT1. **H** Immunofluorescence staining of STAT1 and p-STAT1. Scale bar = 10 μm. **I**, **J** The expression of mRNA and secreted proteins of CXCL9/10. *P < .05, ****P < .0001, *ns* not significant

CD8^+^ T cell skin trafficking requires the IFN-γ-chemokine axis, and keratinocytes are the main source of IFN-γ induced chemokines including CXCL9 and CXCL10 [12]. Hence, we explored the pharmacological role of T-96 in IFN-γ treated keratinocytes. IC50 of T-96 was 12.02 μM in NHEKs (Fig. 5A) and 15.08 μM in HaCaT cells (Additional file 1: Fig. S5A), and the concentration 1 μM was applied for subsequent experiments based on the cell viability assay (Fig. 5B and Additional file 1: Fig. S5B). To obtain the binding proteins of T-96 in IFN-γ stimulated keratinocytes, we performed pull-down assay and mass spectrum analysis, and the proteins with high scores were sorted accordingly for different biochemical functions. The results indicated that T-96 responded to IFN-γ signaling and the protein JAK2 was involved (Fig. 5C). Besides, molecular docking analysis showed that T-96 could interact with JAK2 (Fig. 5D), and formed conventional hydrogen bonds with Ser633 and Asn641, carbon-hydrogen bonds with Tyr637 and Val629, Van der Waals with Thr636, Phe631, Ile559, Leu680, Leu579, Gly632, Phe628 and Lys630, and alkyl interaction with Lys640 and Leu551 (Fig. 5E).

Next, we discovered that pretreatment with T-96 inhibited the phosphorylation of JAK2 in IFN-γ treated keratinocytes at 15 min by flow cytometry (Fig. 5F and Additional file 1: Fig. S5C) and western blot (Additional file 1: Fig. S5D), and Tofa inhibited both JAK1 and JAK2 (Additional file 1: Fig. S5C-F). T-96 also reduced the expression of mRNA, as well as total and phosphorylation proteins of STAT1, which was comparable with that of Tofa, and there was a weak synergistic effect but no statistical difference (Fig. 5G and Additional file 1: Fig. S5G, H). T-96 significantly inhibited the nuclear translocation of STAT1 and the expression of p-STAT1 comparable to the effect of Tofa (Fig. 5H and Additional file 1: Fig. S5I). What’s more, T-96 markedly decreased the mRNA and protein secretion levels of CXCL9 (Fig. 5I) and CXCL10 (Fig. 5J).

**Fig. 6:**
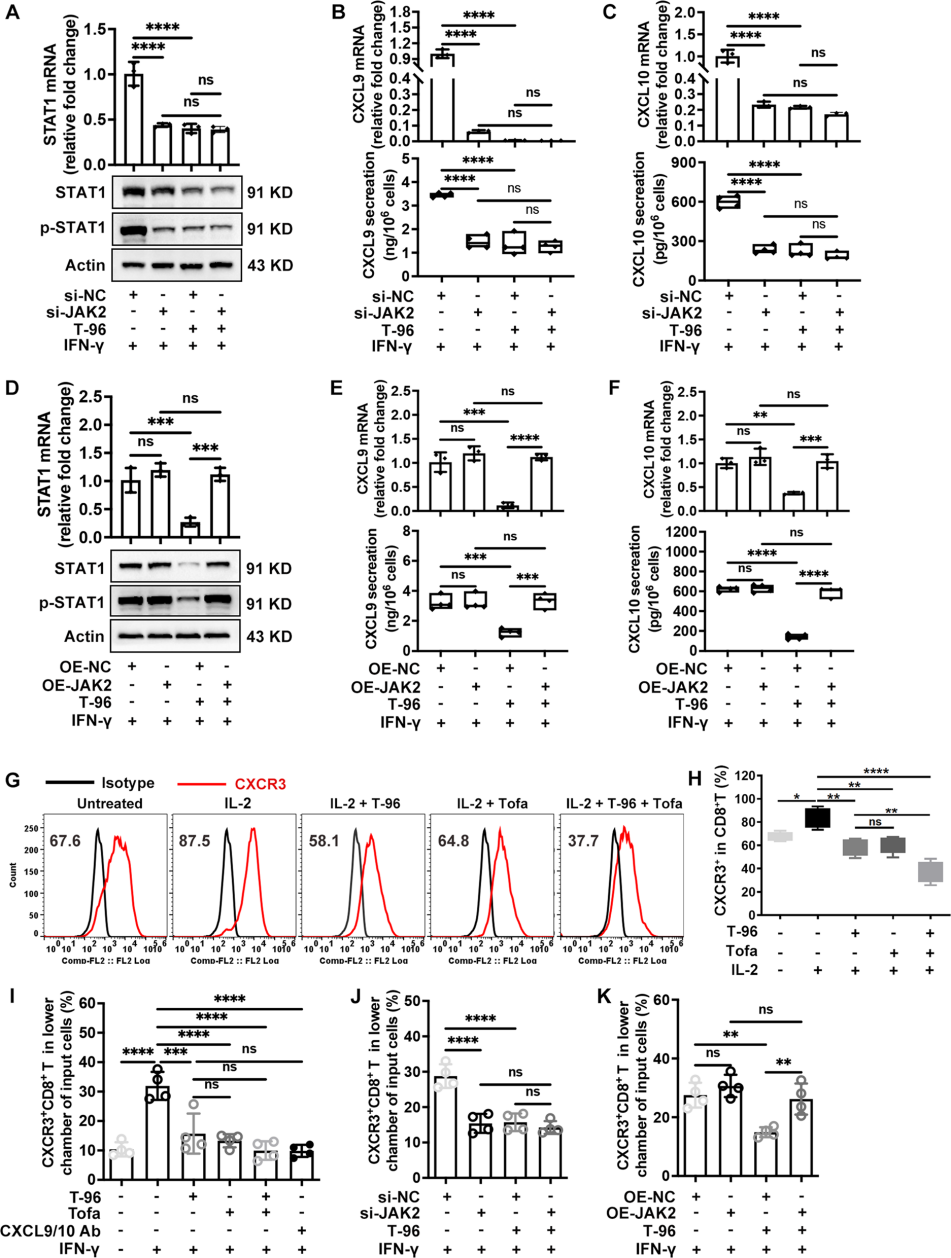
T-96 inhibited STAT1-CXCL9/CXCL10 signaling by targeting JAK2 in IFN-γ stimulated keratinocytes. T-96 downregulated the CXCR3 expression on CD8^+^ T cells and blocked chemotaxis of CXCR3^+^CD8^+^ cells. **A** The mRNA and protein expression of STAT1 upon JAK2 knockdown. **B** The mRNA and secreted proteins of CXCL9 upon JAK2 knockdown. **C** The mRNA and secreted proteins of CXCL10 upon JAK2 knockdown. **D** STAT1 mRNA and protein expression upon JAK2 overexpression. **E** CXCL9 mRNA expression and secreted proteins upon JAK2 overexpression. **F** CXCL10 mRNA expression and secreted proteins upon JAK2 overexpression. **G**, **H** CD8^+^ T cells were cultured in the presence of IL-2 and stimulated with T-96 or Tofa for 48 h. Representative flow cytometric histograms of CXCR3 are shown on left, and the frequency analysis is on right. **I**–**K** The migration of CXCR3^+^CD8^+^ and in 3 h responding to the culture supernatants with indicated treatments in NHEKs. *P < .05, **P < .01, ***P < .001, ****P < .0001. *ns* not significant

Moreover, we evaluated the effects of T-96 following JAK2 knockdown or overexpression. RT-PCR and western blot analysis verified the efficiency of JAK2 knockdown (Additional file 1: Fig. S5J) and overexpression (Additional file 1: Fig. S5K). The results showed that the knockdown of JAK2 significantly decreased the mRNA and protein expressions of STAT1 (Fig. 6A), as well as the mRNA and secreted protein levels of CXCL9 (Fig. 6B) and CXCL10 (Fig. 6C) in IFN-γ treated keratinocytes. And the pretreatment with T-96 didn’t exhibit further significant inhibition on the STAT-CXCL9/10 signaling in the JAK2 knockdown compared to the blank control plasmid group. In addition, T-96 failed to suppress the mRNA and protein expression of STAT1 in the JAK2 overexpression compared to the blank control plasmid group (Fig. 6D). Similarly, the increased mRNA and secreted protein levels of CXCL9 (Fig. 6E) and CXCL10 (Fig. 6F) were not reversed by T-96 when JAK2 overexpression. Taken together, these data suggested that T-96 inhibited STAT1-CXCL9/CXCL10 signaling by targeting JAK2 in IFN-γ stimulated keratinocytes.

### ***T-96 downregulated the cell surface expression of CXCR3 on CD8***^+^***T cells and blocked the chemotaxis ability of CXCR3***^+^***CD8***^+^***T cells***

CXCR3^+^CD8^+^ T cells accumulate in the skin chemoattracted by CXCL9 and CXCL10 contributes to the melanocyte destruction [9, 37]. We asked the question of whether T-96 could influence chemokine receptor CXCR3 expression on CD8^+^ T cells. Using flow cytometry we observed that IL-2 enhanced the CXCR3 membrane expression at 48 h in agreement with another study [38]. Notably, the pretreatment with either T-96 or Tofa decreased the expression of CXCR3, and a synergistic effect was found when simultaneously stimulated with both (Fig. 6G, H). Further, the migration assay showed that the culture supernatants of keratinocytes pretreated with T-96 followed by IFN-γ stimulation blocked the migration of CXCR3^+^CD8^+^ T cells, and the blocking effect is comparable to that of Tofa and neutralization antibodies of CXCL9 and CXCL10 (Fig. 6I). Meanwhile, T-96 did not obviously alter CXCR3^+^CD8^+^ T cell migration upon JAK2 knockdown (Fig. 6J). In contrast, the blocking migration of CXCR3^+^CD8^+^ T cells treated with T-96 was increased following JAK2 overexpression (Fig. 6K). Together, T-96 decreased CXCR3 expression on CD8^+^ T cells from patients with vitiligo and blocked the chemotactic migration of CXCR3^+^CD8^+^ T cells under the CXCL9 and CXCL10 derived from inflamed keratinocytes.

## Discussion

**Fig. 7:**
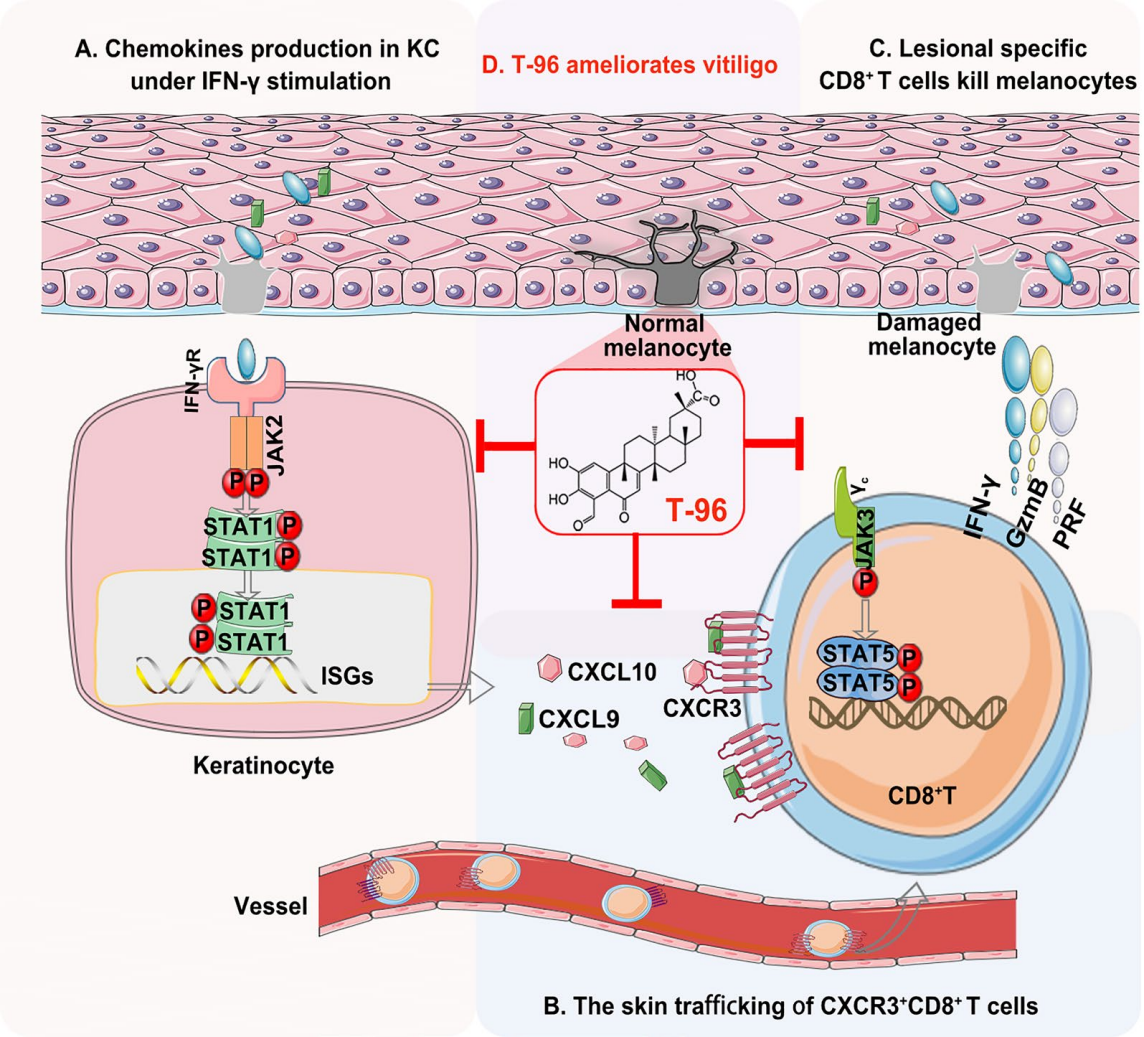
T-96 ameliorated the depigmentation of vitiligo by JAK-STAT signaling. **A** The high levels of IFN-γ in the skin generate abundant chemokine production of keratinocytes via IFN-γ-JAK2-STAT1-CXCL9/10 pathway. **B** CXCL9/10 recognize CXCR3 and result in the recruitment of CXCR3^+^CD8^+^ T cells. **C** Autoreactive CD8^+^ T cells destroy melanocytes using cytotoxic granules granzyme B (GzmB) and perforin (PRF) and establish a high IFN-γ profile. **D** T-96 decreased the production of chemokines in keratinocytes targeting IFN-γ-JAK2-STAT1 signaling, blocked the migration of CXCR3^+^CD8^+^ T cells, suppressed the effector function of CD8^+^ T cells, and finally rescued the loss of melanocytes

Unrestrained activation of the JAK/STAT pathways in CD8^+^ T cells and keratinocytes contributes to vitiligo pathogenesis, making JAKs an attractive target for pharmacologic intervention in vitiligo treatment [39, 40]. In this study, we first clarified that T-96 ameliorated ongoing depigmentation and decreased the infiltrating CD8^+^ T cell numbers in the skin by using our vitiligo mouse model. T-96 suppressed the proliferation, activation, and function of activated CD8^+^ T cells, partly by inhibition of JAK3-STAT5 signaling. Besides, T-96 blocked the migration of CD8^+^ T cells, which was achieved by reducing CXCR3 receptor expression on CD8^+^ T cells and CXCL9 and CXCL10 ligand expression via JAK2-STAT1 pathway in IFN-γ stressed keratinocytes. Our study revealed that T-96 exhibited therapeutic efficacy in our mouse model of vitiligo and had immunosuppressive and migration-blocking effects on CD8^+^ T cells comparable to the pan-JAK inhibitor Tofa (Fig. 7).

T-96, as a novel triterpene extracted from TwHF, has demonstrated therapeutic benefits and lower toxicity for autoimmune disorders [16, 41, 42]. This enabled T-96 to potentially be developed into a drug. According to the genome evolution analysis, TwHF, as a medicinal plant, underwent whole-genome triplication (WGT) and developed the ability to adapt to significantly changed environments [14, 43]. As a traditional herb, TwHF has been widely used to treat various autoimmune diseases including vitiligo [44], psoriasis [45], rheumatoid arthritis [46], and idiopathic IgA nephropathy [47] in China. However, the application of TwHF preparations is limited due to the high toxicity of some ingredients in the whole root and the empirical practice based on traditional medicine theories and doctors’ experiences in clinical settings [45, 48]. Therefore, clarifying the active monomer and molecular pharmacological mechanism of TwHF is critical for treating vitiligo and other autoimmune diseases. Our results showed that the ingredient T-96 was effective in preventing depigmentation in our mouse model of vitiligo. Also, T-96 exhibited no cytotoxicity at 1 μM in CD8^+^ T cells or keratinocytes, consistent with low cytotoxicity reported in other studies [15], 22. Our findings indicated that the component T-96 instead of other TwHF constituents, has the potential for clinical application in managing vitiligo.

The activation and fine-tuning migration of autoreactive CD8^+^ T cells to melanocytes are necessary and sufficient for the progression, exacerbation, and relapse of vitiligo [49]. JAKs are essential enzymes in this process by mediating type I/II cytokines signaling such as IL-2, IL-15, IFN-γ, etc. [50]. IL-2 potently promotes the expansion and generation of effector CD8^+^ T cell after initial T cell receptor (TCR) activation [51]. IFN-γ plays a pivotal role in depigmentation induced by vitiligo [52]. Extracellular binding of IFN-γ activates JAKs, leads to the self-phosphorylation of JAKs, and activates STAT proteins along the inner side of the cellular membrane to form a dimer unit and translocation to the nucleus enabling the transcription of CXCL9 and CXCL10 [53]. These chemokines are important in the recruitment of CXCR3^+^CD8^+^ T cells [54]. In vitiligo skin and peripheral blood mononuclear cells (PBMC) from patients with vitiligo and mouse models of vitiligo, activated CD8^+^ T cells express the chemokine receptor CXCR3. CXCL9 and CXCL10 also show high expression in skin lesions and serum, with CXCL9 recruiting and CXCL10 localizing these cells [12, 55, 56]. In this study, we demonstrated that T-96 inhibited not only the activation and cytotoxic effects of CD8^+^ T cells but also the expression and interaction of CXCR3 and CXCL9/10 between CD8^+^ T cells and IFN-γ-inflamed keratinocytes.

Our research clarified the discordant role of T-96 in regulating JAKs (JAK1, JAK2, and JAK3). JAK proteins interact with different intracellular domains of cytokine receptors and are present in various cell types [50]. Common cytokine-receptor γ-chain (γ_c_, also known as CD132) family of cytokines, including IL-2, IL-4, IL-7, IL-9, IL-15, and IL-21 are essential in driving the development, differentiation, and proliferation of CD8^+^ T cells, through JAK3 interacting with γ_c_, and JAK1 associating with IL-2Rβ, IL-4Rα, IL-7Rα, IL-9Rα, and IL-21Rα [57]. In the current study, we demonstrated that T-96 interacted with JAK3 in the CD8^+^ T cell from vitiligo patients, attenuating IL-2-induced JAK3 phosphorylation. Although IL-2 has been shown to activate multiple STAT proteins encompassing STAT1, STAT3, and STAT5, STAT5 serves as the primary signal transducer downstream of IL-2 signaling [58]. Our findings also revealed that T-96 suppressed STAT5 activation. These data implicated that the pharmacological immunosuppressive action of T-96 on CD8^+^ T cells was ascribed to the blockade of the JAK3-STAT5 signaling cascade. Furthermore, we discovered that T-96 interacted with JAK2 in IFN-γ-stimulated keratinocytes. Notably, T-96 and tofacitinib inhibited the STAT1-CXCL9/10 pathway in inflamed keratinocytes equally well, even though tofacitinib inhibits both JAK1 and JAK2. Strong activation of JAK2 rather than JAK1 was observed in IFN-γ-treated keratinocytes in agreement with a previous study [59], so the hampering of JAK2 is sufficient to prevent the impediment of down-stream signaling in IFN-γ-stressed keratinocytes. The JAK2/STAT1 axis exhibits a predominant role in mediating the responses of keratinocytes to IFN-γ stimulation; therefore, we primarily focused on STAT1 as the canonical transcription factor downstream of JAK2 signaling in IFN-γ-stimulated keratinocytes.

JAK inhibitors (JAKi), such as tofacitinib (a pan JAKi) and ruxolitinib (selectivity for JAK1 and JAK2), have apparently reversed vitiligo in some case reports [34], and ongoing clinical trials [60, 61]. Indeed, the FDA has approved ruxolitinib cream 1.5% for repigmentation in patients with nonsegmental vitiligo. While JAKi can halt disease activity, combination theraphy NBUVB or sunlight is necessary to achieve repigmentation [62, 63]. Currently, it is presumed that next-generation JAKi with either greater selectivity for specific JAKs or organ-selective agents may have improved safety profiles [63–65]. Our study provides a new natural selective JAK2/3 inhibitor T-96, which is more affordable than other JAKi. Comprehensive safety evaluations of these newer agents in larger and longer clinical trials are mandated, considering the complexity of the JAK–STAT pathways and the potential for rare idiosyncratic reactions. Notably, T-96 can also be formulated as ointments, which may decrease the systemic effects and potential adverse reactions. Therefore, future studies are needed to test the efficacy and uncover the underlying mechanisms when used locally.

## Conclusions

Overall, we demonstrate that T-96 is effective in treating vitiligo mediated by inhibiting the effector function and skin homing of CD8 T cells through JAK-STAT signaling. Our study provides evidence that T-96 may be translated for its pharmacological activity against vitiligo and holds promise for future treatment of various inflammatory skin conditions.

## Supplementary Information

**Additional file 1.** Supplementary Figures and Figure Legends.

**Additional file 2.** Supplementary Tables.

## Data Availability

All data associated with this study are available from the corresponding author upon reasonable request.

## Abbreviations

T-96: Demethylzeylasteral
Tofa: Tofacitinib
IL: Interleukin
JAK: Janus kinase
STAT: Signal transducers and activators of transcription
CXCL: C-X-C motif chemokine ligand
CXCR: Chemokine (C-X-C motif) receptor
IFN-γ: Interferon-γ
GzmB: Granzyme B
PRF: Perforin
γc: Common cytokine-receptor γ-chain
NHEKs: Normal human epidermal keratinocytes
PBMC: Peripheral blood mononuclear cell
PBS: Phosphate buffered saline
DAPI: 49-6-Diamidino-2-phenylindole
