# Supplementary material for: Pharmacological inhibition of demethylzeylasteral on JAK-STAT signaling ameliorates vitiligo
Source: J Transl Med. 2023 Jul 4;21:434. doi: 10.1186/s12967-023-04293-2 (PMC10318684; doi:10.1186/s12967-023-04293-2)
Supplement: Supplementary file 1 — Additional file 1. Supplementary Figures and Figure Legends. [file 12967_2023_4293_MOESM1_ESM.doc]

**Supplementary Figures and Figure Legends**

**
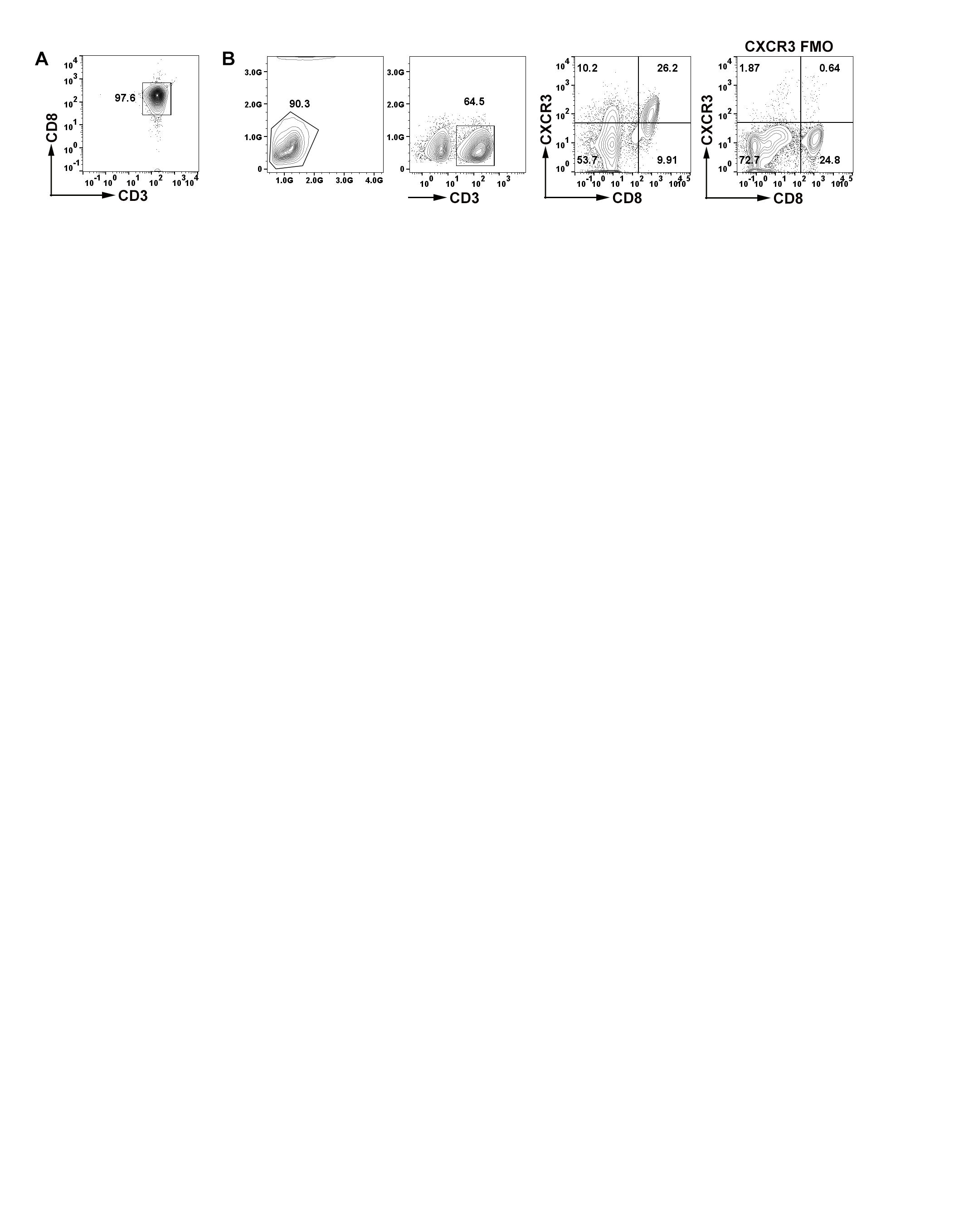
**

**Fig. S1. The purity of** **CD8^+^ T cells by magnetic bead sorting and the gating strategies of** **CXCR3^+^CD8^+^ T cells.** (**A**) Purified CD8^+^ T cells were prepared and negatively selected by magnetic sorting as described in the Methods, and the purity of CD8^+^ T cells was identified over 95% after magnetic bead sorting using flow cytometry analysis. (**B**) The flow cytometry cell sorting strategy of CXCR3^+^CD8^+^ T cells from PBMC of patients with vitiligo. FMO, fluorescence minus one control.

**
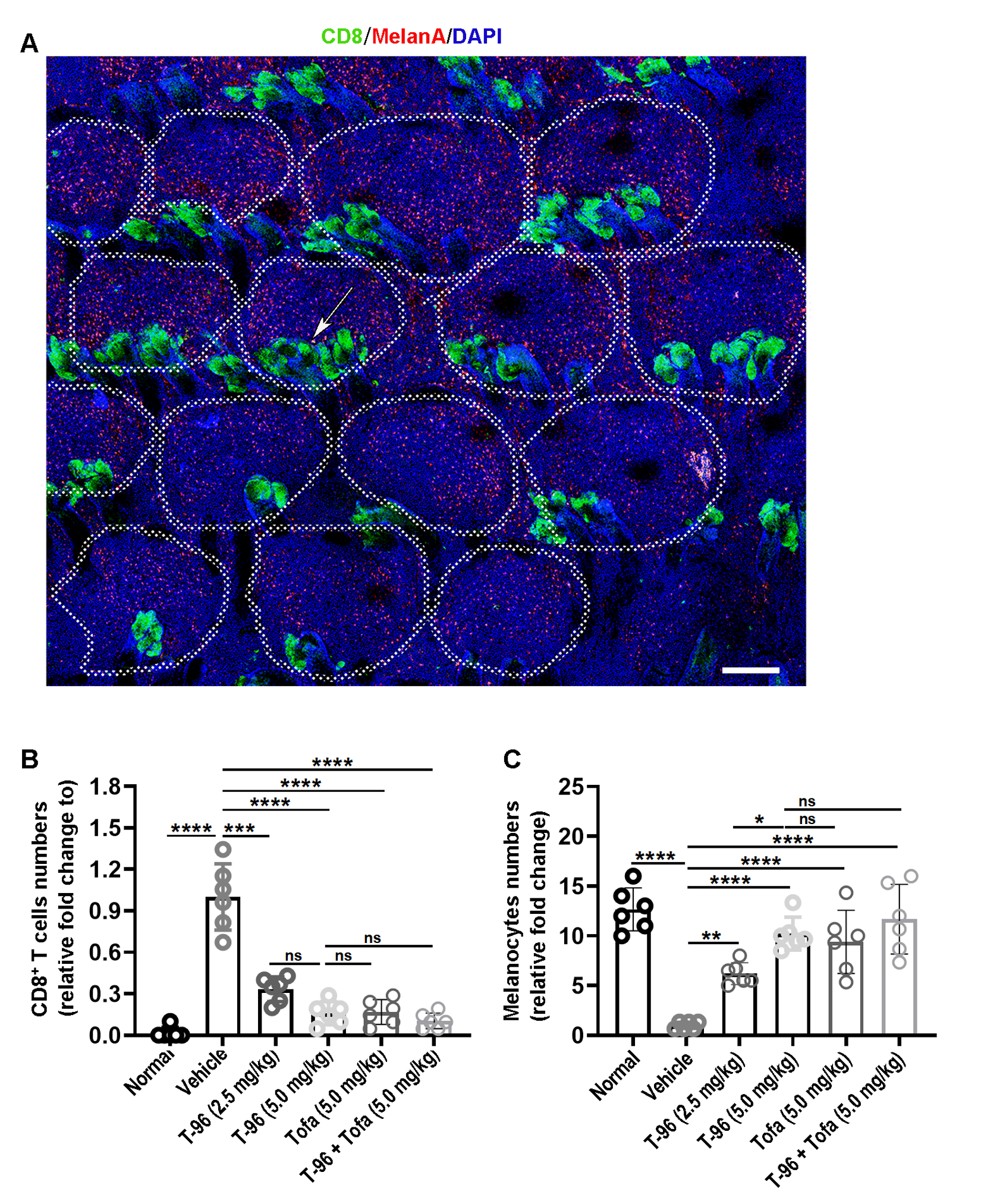
**

**Fig. S2. T-96 ameliorated ongoing depigmentation by inhibiting the CD8^+^ T cell skin infiltration in a mouse model of vitiligo.** (**A**) The whole-mount tail epidermis immunofluorescence staining of normal C57BL/6 mice without the removal of hair follicles and sebaceous glands. The white arrow refers to the sebaceous gland. Scale bar = 200μm. (**B**) Statistical analysis of the number of CD8^+^ T cells for immunofluorescence staining in Figure 1G. (**C**) Statistical analysis of the number of melanocytes for immunofluorescence staining of Figure 1G. ****P* < .001, *****P* < .0001, ns, not significant.

**
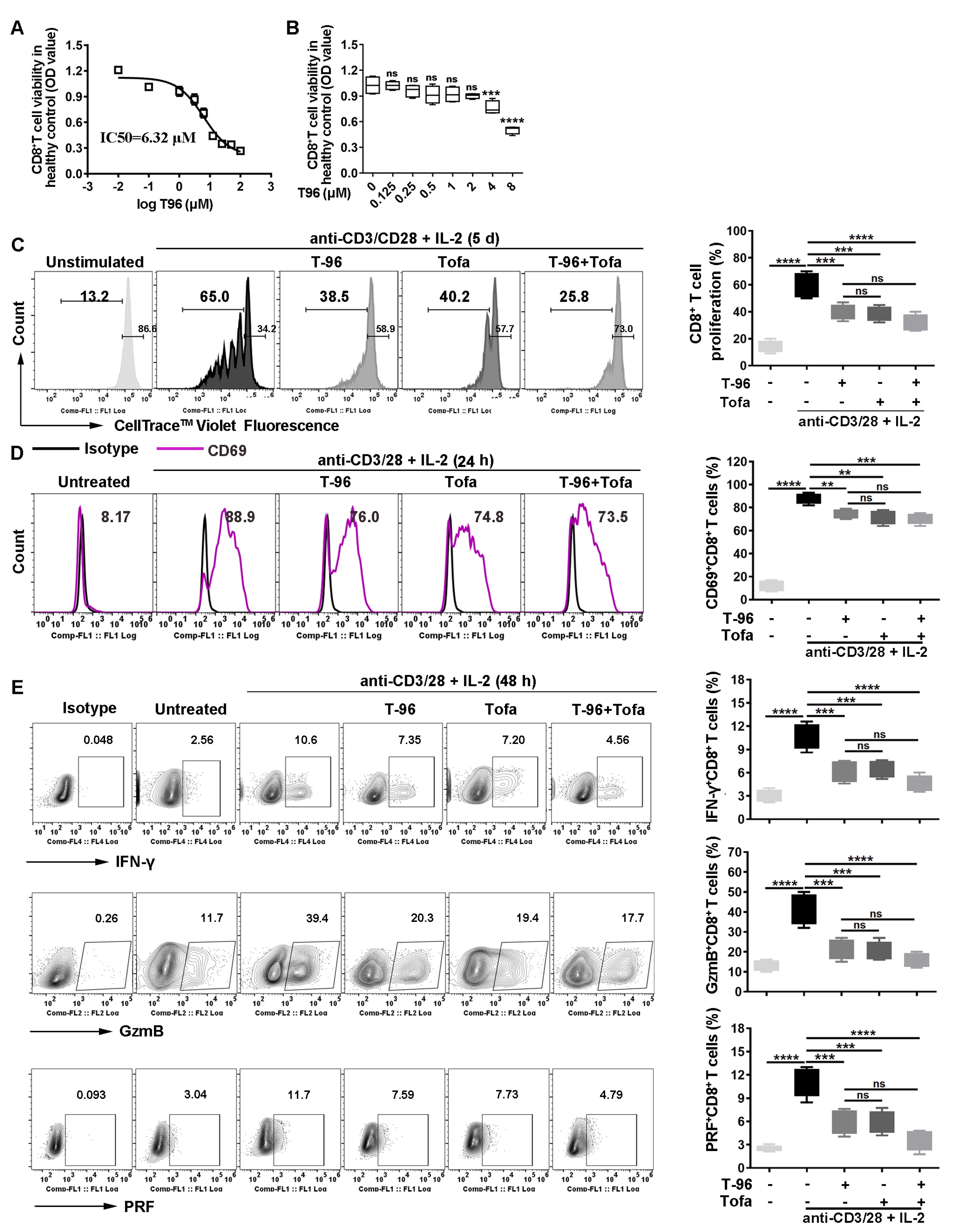
**

**Fig. S3. T-96** **suppressed the** **proliferation, activation, and function of CD8^+^ T cells in** **healthy individuals.** (**A-B**) IC_50_ value and cell viability for CD8^+^ T cells isolated PBMC from healthy controls. Statistical analysis was performed relative to the untreated group. (**C**) Representative proliferation histograms of CFSE-labeled CD8^+^ T cells from healthy controls were shown (left) and the statistical graph (right). (**D-E**) Flow cytometry for the expression of CD69, IFN-γ, GzmB and PRF in CD8^+^ T cells from healthy control, representative examples (left), and frequency analysis (right). ***P*<0.01, ****P*<0.001, *****P*<0.0001, ns, not significant.

**
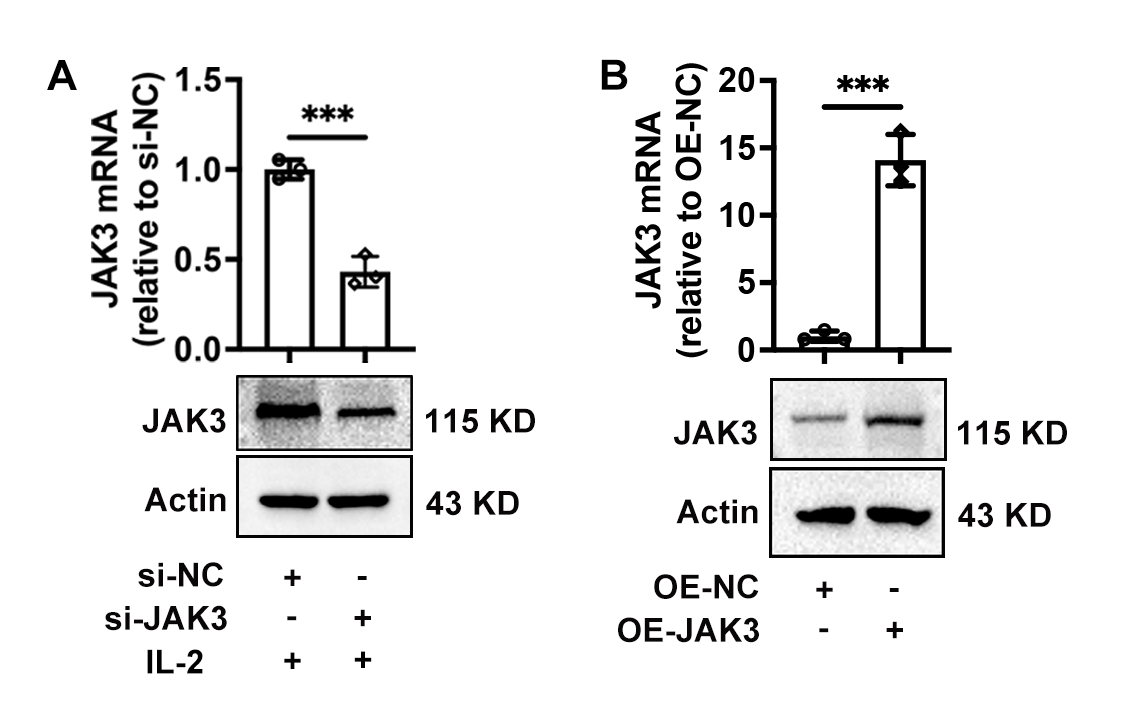
**

**Fig. S4. The efficiency of JAK3 knockdown and overexpression in CD8^+^ T cells.** (**A**) The mRNA and protein level of JAK3 knockdown. (**B**) The mRNA and protein level of JAK3 overexpression. ****P*<0.001.

**
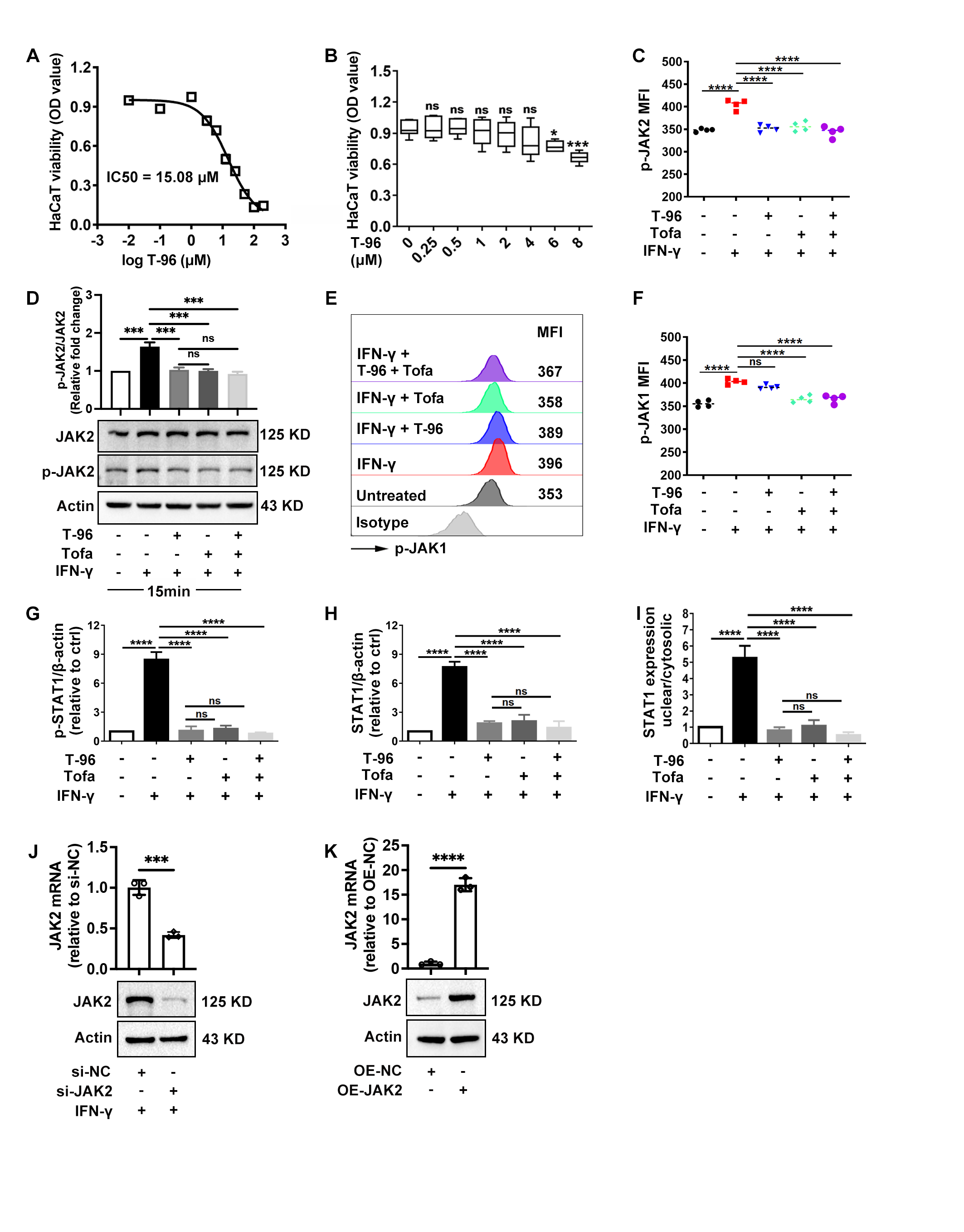
**

**Fig. S5. T-96** **decreased JAK2-STAT1 signaling in IFN-γ treated** **keratinocytes.** (**A-B**) IC_50_ value and cell viability in HaCaT cells. (**C**) The MFI analysis of p-JAK2 in Figure 5F. (**D**) The protein expression of p-JAK2 using western blot in NHEKs. (**E-F**) The expression of p-JAK1 in NHEKs by flow cytometry and the statistical analysis of MFI. (**G-H**) The statistical analysis of the western blot of Figure 5G. (**I**) The analysis of STAT1 immunofluorescence staining of Figure 5H. (**J**) The mRNA and protein level of JAK2 knockdown. (**K**) The mRNA and protein level of JAK2 overexpression. **P* < .05, ****P* < .001, *****P* < .0001, ns, not significant.
