# Supplementary material for: Pharmacological inhibition of demethylzeylasteral on JAK-STAT signaling ameliorates vitiligo
Source: J Transl Med. 2023 Jul 4;21:434. doi: 10.1186/s12967-023-04293-2 (PMC10318684; doi:10.1186/s12967-023-04293-2)
Supplement: Supplementary file 2 — Additional file 2. Supplementary Tables. [file 12967_2023_4293_MOESM2_ESM.doc]

**Supplementary Table 1.** List of primary and second antibodies

| **ANTIBODIES** | **SOURCE** | **IDENTIFIER** |
| --- | --- | --- |
| **Antibodies used for flow cytometry** | | |
| Anti-human IFN-γ [4S.B3] PerCP | Biolegend | Cat# 502524 |
| Anti-human Granzyme B [QA18A28] PE | Biolegend | Cat# 396406 |
| Anti-human CXCR3 [[G025H7](https://www.biolegend.com/en-us/search-results?Clone=G025H7)] PE | Biolegend | Cat#353706 |
| Anti-human CD69 [FN50] FITC | Biolegend | Cat#310904 |
| Anti-human Perforin [B-D48] FITC | Biolegend | Cat#353310 |
| Anti-human p-STAT5 [47] PE | BD | Cat#612567 |
| Anti-human p-JAK3 [JAK3Y980981-E10] PE | Thermo Scientific | Cat#MA537201 |
| Anti-human p-JAK1 [F11] PE | Abwiz Bio | Cat#2412 |
| Anti-human p-JAK2 [PB6] PE | Abwiz Bio | Cat#2457 |
| Anti-mouse IgG1, PerCP | Biolegend | Cat#400147 |
| Anti-Rabbit IgG, PE | NOVUS | Cat#NBP2-24983 |
| Anti-Rat IgG1, κ PE | Biolegend | Cat#400407 |
| Anti-mouse IgG1, κ FITC | Biolegend | Cat#400107 |
| Anti-mouse IgG1, κ PE | Biolegend | Cat#400113 |
| **Antibodies used for western blotting** | | |
| Anti-human JAK3 [5H2] | CST | Cat#5481 |
| Anti-human p-JAK3 [D44E3] | CST | Cat#5031 |
| Anti-human STAT5 [D3N2B] | CST | Cat#25656 |
| Anti-human p-STAT5 [D47E7] | CST | Cat#4322 |
| Anti-human JAK2 [D2E12] | CST | Cat#3230 |
| Anti-human p-JAK2 [E132] | Abcam | Cat#ab32101 |
| Anti-human STAT1 [D1K9Y] | CST | Cat#14994 |
| Anti-human p-STAT1 [D4A7] | CST | Cat#7649 |
| Anti-human β-Actin [8H10D10] | CST | Cat#3700 |
| **Antibodies used for immunofluorescence** | | |
| Anti-human STAT1 | Abcam | Cat# ab281999 |
| Anti-human p-STAT1 [D4A7] | CST | Cat#7649 |
| Goat-anti- Mouse IgG FITC | Abcam | Cat#ab6785 |
| Goat-anti-Rabbit IgG cy3 | Abcam | Cat#ab6939 |
| **Antibodies used for Whole-mount tail staining** | | |
| Anti-CD8 alpha [YTS169.4] | Abcam | Cat#ab22378 |
| Anti-mouse MelanA [M2-9E3] | Abcam | Cat#ab187369 |
| Goat-anti-Mouse IgG cy3 | Abcam | Cat#ab97035 |
| Goat-Anti-Rat IgG H&L FITC | Abcam | Cat#ab6840 |

**Supplementary Table 2.** Quantitative real-time PCR primers sequences

| Gene | Forward (5’ to 3’) | Reverse (5’ to 3’) |
| --- | --- | --- |
| JAK2 | CTTTCAGAGCCATCATACGAG | TTTTTTACAGCGACCACCT |
| JAK3 | CAGCTGGGCAAGGGCAACTTT | ACACCACGATACTTGACAATG |
| STAT1 | ATGGCAGTCTGGCGGCTGAATT | CCAAACCAGGCTGGCACAATTG |
| CXCL9 | TCTAAACCCAGATTCAGCA | CTCCTTTGGAATGATAGCG |
| CXCL10 | CCTCCAGTCTCAGCACCAT | AAATTGGCTTGCAGGAATA |
| Actin | CGTGCCGCCTGGAGAAAC | AGTGGGAGTTGCTGTTGAAGTC |

**Supplementary Table 3.** Clinical characteristics of vitiligo patients

| Identifier | Gender | Age  (years) | White patches area (BSA) | Classification | Disease stages | Disease duration (months) | Associated  Diseases |
| --- | --- | --- | --- | --- | --- | --- | --- |
| Vit 01 | Female | 31 | 11.7 | Nonsegmental | progressive | 180 | none |
| Vit 02 | Female | 26 | 1.2 | Nonsegmental | progressive | 110 | none |
| Vit 03 | male | 37 | 6.2 | Nonsegmental | progressive | 24 | none |
| Vit 04 | male | 33 | 1.4 | Nonsegmental | progressive | 6 | none |
| Vit 05 | male | 43 | 6.5 | Nonsegmental | Progressive | 80 | none |
| Vit 06 | male | 42 | 2.7 | Nonsegmental | Progressive | 48 | none |
| Vit 07 | male | 32 | 3.2 | Nonsegmental | Progressive | 120 | none |
| Vit 08 | male | 38 | 2.0 | Nonsegmental | Progressive | 36 | none |
| Vit 09 | male | 48 | 6.8 | Nonsegmental | Progressive | 50 | none |
| Vit 10 | male | 16 | 7.5 | Nonsegmental | Progressive | 12 | none |
| Vit 11 | male | 46 | 8.1 | Nonsegmental | progressive | 120 | none |
| Vit 12 | Female | 31 | 1.4 | Nonsegmental | progressive | 20 | none |
| Vit 13 | male | 32 | 3.5 | Nonsegmental | progressive | 26 | none |
| Vit 14 | Female | 40 | 1.8 | Nonsegmental | progressive | 84 | none |
| Vit 15 | male | 41 | 1.3 | Nonsegmental | progressive | 180 | none |
| Vit 16 | Female | 48 | 1.8 | Nonsegmental | progressive | 30 | none |
| Vit 17 | Female | 41 | 5.0 | Nonsegmental | progressive | 30 | none |
| Vit 18 | Female | 20 | 3.5 | Nonsegmental | progressive | 60 | none |
| Vit 19 | Female | 23 | 1.6 | Nonsegmental | progressive | 72 | none |
| Vit 20 | male | 33 | 1.7 | Nonsegmental | progressive | 180 | none |
| Vit 21 | male | 39 | 5.5 | Nonsegmental | progressive | 120 | none |
| Vit 22 | Female | 26 | 4.0 | Nonsegmental | progressive | 20 | none |
| Vit 23 | male | 39 | 7.2 | Nonsegmental | progressive | 84 | none |
| Vit 24 | male | 44 | 4.8 | Nonsegmental | progressive | 180 | none |
| Vit 25 | Female | 31 | 5.0 | Nonsegmental | progressive | 72 | none |
| Vit 26 | male | 46 | 1.5 | Nonsegmental | progressive | 120 | none |
| Vit 27 | Female | 40 | 2.5 | Nonsegmental | progressive | 30 | none |
| Vit 28 | Female | 28 | 2.5 | Nonsegmental | progressive | 20 | none |
| Vit 29 | Female | 31 | 3.4 | Nonsegmental | progressive | 20 | none |
| Vit 30 | Female | 38 | 5.0 | Nonsegmental | progressive | 90 | none |
| Vit 31 | male | 28 | 1.8 | Nonsegmental | progressive | 24 | none |
| Vit 32 | male | 33 | 2.5 | Nonsegmental | progressive | 7 | none |
